# Analytical Modeling and Data-Driven Uncertainty Analysis of the Vibration Response of Partially Liquid-Filled Rotors Under Lateral Excitation

**DOI:** 10.3390/ma19091728

**Published:** 2026-04-24

**Authors:** Hongyun Sun, Xinjie Bai, Xinqi Li, Hongyuan Zhang, Yang Shao, Huiqun Yuan

**Affiliations:** 1 School of Automotive and Transportation, Shenyang Ligong University, Shenyang 110159, China; 17832875307@163.com (X.B.); 15715380361@163.com (X.L.); zhy_sylu@163.com (H.Z.);; 2 College of Sciences, Northeastern University, Shenyang 110819, China

**Keywords:** partially liquid-filled rotor, lateral excitation, fluid–structure interaction, surrogate modeling, uncertainty quantification

## Abstract

Partially liquid-filled rotor systems subjected to lateral excitation exhibit pronounced fluid–structure interaction, leading to complex and highly sensitive vibration responses. To enable efficient probabilistic prediction under parametric uncertainty, this study develops a deterministic–data-driven framework for a rigid hollow rotor partially filled with liquid. Based on small-perturbation flow theory, the liquid-induced feedback forces are analytically derived and incorporated into the coupled rotor–liquid dynamic equations, yielding a closed-form steady-state solution. The results reveal that lateral excitation in one direction induces coupled vibration in the orthogonal direction, resulting in an elliptical whirl trajectory of the rotor center. The vibration characteristics depend jointly on excitation frequency and rotor angular velocity, and for a given angular velocity, two critical excitation frequencies are identified at which the response amplitude increases sharply. Surrogate models based on a backpropagation neural network (BPNN) and a support vector machine (SVM) are constructed and validated, with the BPNN demonstrating superior predictive accuracy. Uncertainty analysis further shows that the maximum vibration amplitude exhibits asymmetric, non-Gaussian distributions even under normally distributed inputs, and excessive amplification may occur beyond certain uncertainty levels. The proposed framework provides a robust tool for probabilistic vibration assessment and uncertainty-informed design of partially liquid-filled rotor systems.

## 1. Introduction

Partially liquid-filled rotors (PLFRs) occur in many rotating machines where a hollow rotor or drum contains a liquid layer or an annular liquid ring, such as rotating drums and separators, centrifugal devices, and rotors equipped with liquid balancing rings [1,2,3]. In these systems, the internal liquid is not a passive payload. When the rotor experiences complex motion, the liquid redistributes and generates a disturbance pressure field that produces additional forces acting back on the rotor [4,5,6]. This fluid–rotor interaction may change the effective inertia and damping, introduce cross-coupled terms, and reshape the steady-state whirl orbit. Accurate prediction of the steady-state forced response of PLFR systems is therefore essential for vibration control, reliable operation, and robust rotor design [7,8,9].

A considerable body of research has investigated PLFR dynamics, historically with strong emphasis on self-excited instabilities and subsynchronous whirling. Early analytical studies established fundamental mechanisms by which the internal liquid can destabilize the rotor over specific speed ranges through phase-lag and cross-coupled fluid forces [10,11,12]. Later developments incorporated viscous effects and more refined stability analyses, showing that viscosity and free-surface behavior can markedly influence instability thresholds and whirl characteristics [13,14,15,16]. In parallel, experiments and application-driven studies on liquid balancing devices demonstrated that liquid redistribution may either reduce or amplify vibration depending on the operating regime and parameter combinations [17,18,19]. More recent work has expanded toward more realistic configurations, including flexible rotors, thermal environments, functionally graded density fluid and structurally nonuniform rotors (e.g., functionally graded hollow rotors), thereby improving the relevance to practical machines and revealing additional couplings between structural properties and fluid-induced forces [20,21,22].

Despite the substantial progress made in understanding the dynamics of partially liquid-filled rotors, two issues remain insufficiently addressed. First, much of the existing literature is primarily concerned with self-excited instability and subsynchronous whirl, whereas comparatively fewer studies provide tractable formulations for the forced lateral response of PLFR systems under prescribed external excitation [23,24,25]. In many practical applications, however, the primary engineering concern is not only whether instability occurs, but also the amplitude and orbit of the steady-state response under lateral disturbances. A physics-based formulation capable of explicitly representing liquid-induced feedback forces is therefore still needed for efficient forced-response prediction [26,27,28,29].

Second, most available PLFR analyses are deterministic, while practical rotor systems inevitably involve uncertainties in filling condition, support properties, and operating parameters [30,31]. Variations in liquid-fill ratio, excitation frequency, rotational speed, and support characteristics may significantly alter the vibration amplitude, distort the shaft-center orbit, and shift resonance-like regions in ways that cannot be captured by nominal-parameter predictions alone [32,33,34,35]. Consequently, probabilistic vibration assessment is essential for understanding response variability and for identifying the conditions under which uncertainty may trigger excessive response amplification [36,37,38,39,40].

Uncertainty quantification and global sensitivity analysis provide a principled way to address this issue by propagating uncertain inputs through a physics-based model and identifying the parameters that dominate output variability [41,42]. However, direct Monte Carlo simulation may become computationally demanding when repeated evaluations are needed, especially if the model is extended to include more realistic fluid effects or nonlinearities [43,44]. This motivates the use of surrogate models as efficient approximations of the original input–output relationship [45]. Although response surface methods, neural networks, and support vector machines have been widely applied in nonlinear regression and uncertainty analysis, their application to forced-response prediction of partially liquid-filled rotors, especially within a physics-informed probabilistic framework, remains limited. In particular, there is still insufficient guidance on how to combine an analytically derived rotor–liquid model with surrogate-assisted uncertainty propagation to reveal the statistical characteristics of maximum vibration amplitudes and the dominant uncertainty drivers.

Motivated by these gaps, this study develops a deterministic–data-driven framework for vibration prediction and uncertainty quantification of a partially liquid-filled rotor subjected to lateral harmonic excitation. A rigid hollow rotor symmetrically supported by flexible bearings is considered. Based on small-perturbation flow theory, the disturbance pressure field and liquid-induced feedback forces are analytically derived and incorporated into the coupled rotor–liquid dynamic equations, yielding a steady-state solution. On this basis, key nondimensional parameters are treated as uncertain, and two surrogate models, namely a backpropagation neural network (BPNN) and a support vector machine (SVM), are constructed and comparatively assessed. The validated surrogate model is subsequently used for Monte Carlo simulation and global sensitivity analysis.

The contributions of this work are as follows. First, a physics-based analytical model is established for the lateral forced response of partially liquid-filled rotors, explicitly accounting for liquid-induced feedback forces in the coupled equations of motion. Second, a deterministic–data-driven uncertainty quantification framework is developed by integrating analytical modeling, surrogate learning, Monte Carlo simulation, and sensitivity analysis. Third, the study provides new insight into the vibration behavior of PLFR systems by revealing cross-coupled elliptical whirl motion, critical excitation conditions, and stochastic response characteristics under parameter uncertainty.

The remainder of this paper is organized as follows. Section 2 develops the dynamic model of the partially liquid-filled rotor, including the formulations of fluid and structural dynamics. Section 3 investigates the dynamic response characteristics and examines the influence of key nondimensional parameters. Section 4 presents the data-driven surrogate modeling and uncertainty quantification framework, including surrogate construction, validation, uncertainty propagation, and global sensitivity analysis. Finally, Section 5 summarizes the main conclusions of the study.

## 2. Dynamic Modeling of the Partially Liquid-Filled Rotor

### 2.1. Fluid Dynamics

**Figure 1:**
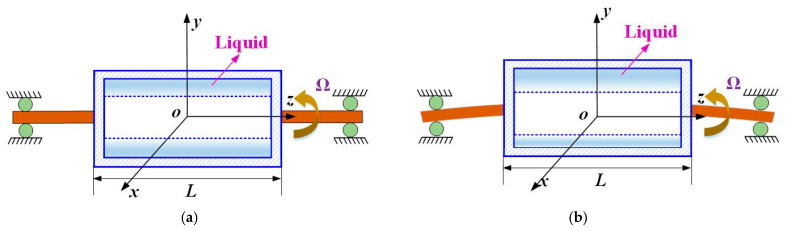
Schematic of a partially liquid-filled rotor: (**a**) steady spin; (**b**) small perturbation motion.

Figure 1 illustrates a rigid hollow rotor partially filled with liquid. The rotor is characterized by a length *L*, a mass $m_{R}$, and an inner radius *a*. It is symmetrically mounted at both ends on a flexible shaft, which is supported by bearings.

In this study, the internal liquid is modeled as an incompressible, inviscid fluid with constant density. The governing equations are derived from the Navier–Stokes equations, which reduce to the Euler equations under the inviscid assumption [10,46,47]. The linearized formulation enables analytical tractability and efficient evaluation of the coupled rotor–liquid dynamics, while capturing the dominant inertia-driven fluid–structure interaction. However, viscous effects, nonlinear free-surface deformation, and large-amplitude fluid motion are neglected. Therefore, the model is valid primarily under small perturbation and low-viscosity conditions, where inertial effects dominate the fluid response.

The bearings are modeled as ideal supports, in which the support forces are proportional to displacement and velocity through constant stiffness and damping coefficients, while nonlinear effects such as clearance and lubricant dynamics are neglected. These modeling assumptions are widely adopted in rotor dynamics and fluid–structure interaction studies and provide an analytically tractable framework while retaining the essential physics governing the coupled rotor–liquid system.

**Figure 2:**
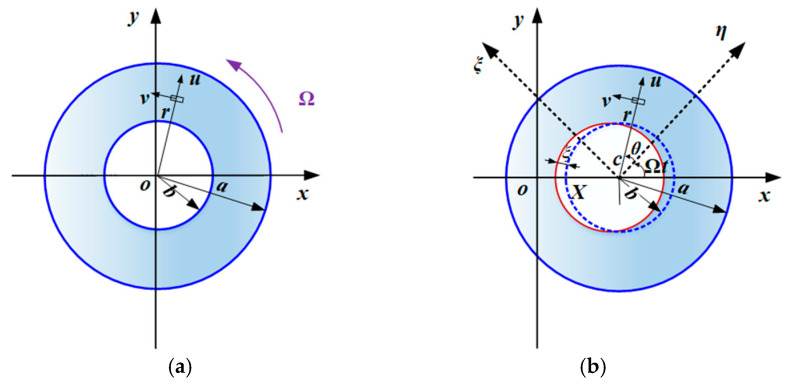
Schematic diagram of partially liquid-filled rotor motion: (**a**) steady spin; (**b**) small perturbation motion.

During undisturbed steady rotation at an angular velocity Ω, the liquid in the cavity redistributes under centrifugal forces and forms a uniform annular layer that adheres to the inner wall of the rotor, thereby constituting a hollow liquid column co-rotating with the rotor [4,47,48]. The corresponding configuration is schematically shown in Figure 1a and Figure 2a, where the inner radius of the liquid column is denoted by *b*.

When the rotor is subjected to a lateral displacement excitation, the cavity liquid does not, in general, follow the rotor in a synchronous perturbation [20,25,27]. Instead, the free surface deforms and the liquid layer becomes circumferentially nonuniform, as illustrated in Figure 2b. Such perturbation-induced redistribution of the internal liquid gives rise to additional hydrodynamic forces acting on the rotor and modifies the coupled dynamic response [9,28]. Therefore, in order to analyze the vibration behavior of the rotor, it is necessary to first characterize the perturbed motion of the cavity liquid and the resulting fluid-induced forces.

Assume that the rigid rotor is subjected to a lateral displacement excitation $X\left(t\right)=\delta \cos\omega t\;(\delta \ll a)$ in the *x*-direction. To evaluate the perturbed pressure acting on the rotor, an inertial (fixed) Cartesian coordinate system o−xyz is defined, with the origin o located at the midpoint of the line connecting the centers of the two end bearings, i.e., on the rotor axis in the stationary (unperturbed) configuration. A body-fixed convected coordinate system c−ηξζ attached to the rotor is further introduced, together with a cylindrical coordinate system c−rθζ, where *c* denotes the centroid of the rotor cylinder after perturbation.

In the rotor cross-sectional plane perpendicular to the stationary axis, the position of an arbitrary fluid point inside the rotor is specified by the polar coordinates in the moving frame. Let $r_{0}$ and $\theta_{0}$ denote theunit vectors in the orthogonal polar coordinate system, respectively. The relative perturbation velocity of the fluid point then be written as

(1) $$v_{r}=ur_{0}+v\theta_{0}$$

The pressure at this point comprises contributions from the centrifugal acceleration of the fluid and a small perturbed pressure, which can be written as [49]

(2) $$P=\frac{1}{2}\rho \Omega^{2}\pi \left(r^{2}-b^{2}\right)+\bar{p}$$

where $\bar{p}=\bar{p}\left(r,\theta \right)$ denotes the small perturbed pressure.

Assuming small-amplitude fluid motion and neglecting viscosity, the perturbation dynamics in the rotating frame are described by the linearized Navier–Stokes equations as follows [50]:

(3) $$\left\{\begin{array}{l} \frac{\partial u}{\partial t}-2\Omega v=-\frac{1}{\rho }\frac{\partial \bar{p}}{\partial r}+\delta \omega^{2}\cos\omega t\cos(\theta +\Omega t)\\ \frac{\partial v}{\partial t}+2\Omega u=-\frac{1}{\rho }\frac{\partial \bar{p}}{r\partial \theta }-\delta \omega^{2}\cos\omega t\sin(\theta +\Omega t) \end{array}\right.$$

And the continuity equation of the fluid is

(4) $$\frac{\partial \left(ru\right)}{\partial r}+\frac{\partial v}{\partial \theta }=0$$

Combining Equations (3) and (4) with trigonometric identities yields

(5) $$\left\{\begin{array}{l} \frac{\partial u}{\partial t}-2\Omega v=-\frac{1}{\rho }\frac{\partial \bar{p}}{\partial r}+\delta \omega^{2}\left[\cos\left(\theta +\Omega t+\omega t\right)+\cos\left(\theta +\Omega t-\omega t\right)\right]/2\\ \frac{\partial v}{\partial t}+2\Omega u=-\frac{1}{\rho r}\frac{\partial \bar{p}}{\partial \theta }-\delta \omega^{2}\left[\sin\left(\theta +\Omega t+\omega t\right)+\sin\left(\theta +\Omega t-\omega t\right)\right]/2\\ \frac{\partial u}{\partial r}+\frac{\partial v}{r\partial \theta }+\frac{u}{r}=0 \end{array}\right.$$

To solve Equation (5), the associated boundary conditions must be specified. One boundary is the free surface, whose perturbed equation can be written as

(6) r=b+ξ(θ,t)

where ξ(θ,t) denotes the free-surface displacement (perturbation). Substituting this equation into Equation (2) and enforcing the dynamic boundary condition *P* = 0 at the free surface yields [20]

(7) $$\rho \Omega^{2}b\xi +{\left.\bar{p}\right|}_{r=b}=0$$

Since the radial velocity at the free surface is given by

(8) $${\left.u\right|}_{r=b}=\frac{\partial \xi }{\partial t}$$

Substituting this expression into the differentiated form of Equation (7) yields the free-surface boundary condition:

(9) $${\left.\frac{\partial p_{1}}{\partial t}\right|}_{r=b}=-\rho \Omega^{2}b\frac{\partial \xi }{\partial t}=-\rho \Omega^{2}b{\left.u\right|}_{r=b}$$

On the fluid–structure interface at the rotor inner wall, the boundary condition for the flow field can be written as

(10) $${\left.u\right|}_{r=a}=0$$

By assuming a harmonic solution, u, v, $\bar{p}$ can be written in the form

(11) $$\left\{\begin{array}{l} u={\bar{u}}_{1}(r)\sin\left(\theta +\Omega t+\omega t\right)+{\bar{u}}_{2}(r)\sin\left(\theta +\Omega t-\omega t\right)\\ v={\bar{v}}_{1}(r)\cos\left(\theta +\Omega t+\omega t\right)+{\bar{v}}_{2}\cos\left(\theta +\Omega t-\omega t\right)\\ \bar{p}={\bar{p}}_{1}(r)\cos\left(\theta +\Omega t+\omega t\right)+{\bar{p}}_{2}(r)\cos\left(\theta +\Omega t-\omega t\right) \end{array}\right.$$

By virtue of the superposition principle for linear systems, the contributions associated with $\cos\left(\theta +\Omega t+\omega t\right)$ and $\cos\left(\theta +\Omega t-\omega t\right)$ in Equation (5) can be treated separately. We first solve for ${\bar{u}}_{1}$, ${\bar{v}}_{1}$, and ${\bar{p}}_{1}$. Substituting Equation (11) into the first two equations in Equation (5) yields

(12) $$\left\{\begin{array}{l} \sigma_{1}{\bar{u}}_{1}-2\Omega {\bar{v}}_{1}=-\frac{1}{\rho }\frac{d{\bar{p}}_{1}}{dr}+\frac{1}{2}\delta \omega^{2}\\ 2\Omega {\bar{u}}_{1}-\sigma_{1}{\bar{v}}_{1}=\frac{1}{\rho }\frac{{\bar{p}}_{1}}{r}-\frac{1}{2}\delta \omega^{2} \end{array}\right.$$

where $\sigma_{1}=\Omega +\omega$, yielding

(13) $$\left\{\begin{array}{l} {\bar{u}}_{1}=\frac{1}{4\Omega^{2}-\sigma_{1}^{2}}\left[\frac{1}{\rho }\left(\sigma_{1}\frac{d{\bar{p}}_{1}}{dr}+2\Omega \frac{{\bar{p}}_{1}}{r}\right)-\frac{1}{2}(2\Omega +\sigma_{1})\delta \omega^{2}\right]\\ {\bar{v}}_{1}=\frac{1}{4\Omega^{2}-\sigma_{1}^{2}}\left[\frac{1}{\rho }\left(2\Omega \frac{d{\bar{p}}_{1}}{dr}+\sigma_{1}\frac{{\bar{p}}_{1}}{r}\right)-\frac{1}{2}(2\Omega +\sigma_{1})\delta \omega^{2}\right] \end{array}\right.$$

Substituting Equation (11) into the third equation of Equation (5) yields

(14) $${\bar{u}}_{1}\sin(\sigma_{1}t+\theta )+r\frac{\partial {\bar{u}}_{1}}{\partial r}\sin(\sigma_{1}t+\theta )+{\bar{v}}_{1}[-\sin(\sigma_{1}t+\theta )]=0$$

yielding

(15) $${\bar{u}}_{1}+r\frac{\partial {\bar{u}}_{1}}{\partial r}-{\bar{v}}_{1}=0$$

Substituting Equation (13) into Equation (15) yields

(16) $$r^{2}\frac{d^{2}{\bar{p}}_{1}}{dr^{2}}+r\frac{d{\bar{p}}_{1}}{dr}-{\bar{p}}_{1}=0$$

The solution of Equation (16) is given by

(17) $${\bar{p}}_{1}=c_{1}r+\frac{c_{2}}{r}$$

Substituting Equation (17) into the first of Equation (13) gives

(18) $${\bar{u}}_{1}=\frac{1}{4\Omega^{2}-\sigma_{1}^{2}}\left[\frac{2\Omega +\sigma_{1}}{\rho }c_{1}+\frac{2\Omega -\sigma_{1}}{\rho }\frac{c_{2}}{r_{2}}-\frac{1}{2}\delta (2\Omega +\sigma_{1})\omega^{2}\right]$$

Substituting Equations (17) and (18) into the boundary conditions in Equations (9) and (10) yields

(19) $$\left\{\begin{array}{c} \left(2\Omega +\sigma_{1}\;)c_{1}+(2\Omega -\sigma_{1})\frac{c_{2}}{a^{2}}=\frac{1}{2}\delta \rho \omega^{2}(2\Omega +\sigma_{1}\right)\\ \left(\sigma_{1}-\frac{\Omega^{2}}{2\Omega -\sigma_{1}\;}\right)c_{1}+\left(\sigma_{1}-\frac{\Omega^{2}}{2\Omega +\sigma_{1}\;}\right)\frac{c_{2}}{b^{2}}=-\frac{1}{2}\frac{\delta \rho \omega^{2}\Omega^{2}}{2\Omega -\sigma_{1}} \end{array}\right.$$

Solving Equation (19) yields

(20) $$\left(\begin{array}{l} c_{1}\\ c_{2} \end{array}\right)=\frac{1}{2}\frac{\delta \rho \omega^{2}a^{2}b^{2}}{\left(a^{2}-b^{2}\right)\left(2\Omega \sigma_{1}-\Omega^{2}+\sigma_{1}^{2}\kappa \right)}\left(\begin{array}{c} \frac{\sigma (2\Omega +\sigma_{1})}{b^{2}}-\frac{a^{2}-b^{2}}{a^{2}b^{2}}\Omega^{2}\\ -\sigma_{1}(2\Omega +\sigma_{1}) \end{array}\right)$$

where

(21) $$\kappa =\frac{a^{2}+b^{2}}{a^{2}-b^{2}}$$

Substituting Equation (20) into Equation (17) yields

(22) $${\bar{p}}_{1}(r)=\frac{1}{2}\frac{\delta \rho \omega^{2}a^{2}b^{2}}{\left(a^{2}-b^{2}\right)\left(2\Omega \sigma_{1}-\Omega^{2}+\sigma_{1}^{2}\kappa \right)}\left\{\left[\frac{\sigma_{1}(2\Omega +\sigma_{1})}{b^{2}}-\frac{a^{2}-b^{2}}{a^{2}b^{2}}\Omega^{2}\right]r-\frac{\sigma_{1}(2\Omega +\sigma_{1})}{r}\right\}$$

Likewise, it follows that

(23) $$\left\{\begin{array}{l} {\bar{u}}_{2}=\frac{1}{4\Omega^{2}-\sigma_{2}^{2}}\left[\frac{1}{\rho }\left(\sigma_{2}\frac{d{\bar{p}}_{2}}{dr}+2\Omega \frac{{\bar{p}}_{2}}{r}\right)-\frac{1}{2}(2\Omega +\sigma_{2})\delta \omega^{2}\right]\\ {\bar{v}}_{2}=\frac{1}{4\Omega^{2}-\sigma_{2}^{2}}\left[\frac{1}{\rho }\left(2\Omega \frac{d{\bar{p}}_{2}}{dr}+\sigma_{2}\frac{{\bar{p}}_{2}}{r}\right)-\frac{1}{2}(2\Omega +\sigma_{2})\delta \omega^{2}\right] \end{array}\right.$$

where $\sigma_{2}=\Omega -\omega$; thus,

(24) $$\left(\begin{array}{l} c_{3}\\ c_{4} \end{array}\right)=\frac{1}{2}\frac{\delta \rho \omega^{2}a^{2}b^{2}}{\left(a^{2}-b^{2}\right)\left(2\Omega \sigma_{2}-\Omega^{2}+\sigma_{2}^{2}\kappa \right)}\left(\begin{array}{c} \frac{\sigma_{2}(2\Omega +\sigma_{2})}{b^{2}}-\frac{a^{2}-b^{2}}{a^{2}b^{2}}\Omega^{2}\\ -\sigma_{2}(2\Omega +\sigma_{2}) \end{array}\right)$$

The following expression for ${\bar{p}}_{2}(r)$ is obtained:

(25) $${\bar{p}}_{2}(r)=\frac{1}{2}\frac{\delta \rho \omega^{2}a^{2}b^{2}}{\left(a^{2}-b^{2}\right)\left(2\Omega \sigma_{2}-\Omega^{2}+\sigma_{2}^{2}\kappa \right)}\left\{\left[\frac{\sigma_{2}(2\Omega +\sigma_{2})}{b^{2}}-\frac{a^{2}-b^{2}}{a^{2}b^{2}}\Omega^{2}\right]r-\frac{\sigma_{2}(2\Omega +\sigma_{2})}{r}\right\}$$

Substituting Equations (22) and (25) into Equation (11) yields the perturbed pressure as

(26) p¯=12δρω2a2b2a2−b22Ωσ1−Ω2+σ12κcosσ1t+θ σ1(2Ω+σ1)b2−a2−b2a2b2Ω2r−σ1(2Ω+σ1)r +12δρω2a2b2a2−b22Ωσ2−Ω2+σ22κcosσ2t+θ σ2(2Ω+σ2)b2−a2−b2a2b2Ω2r−σ2(2Ω+σ2)r

Substituting Equation (26) into Equation (2) and letting r=a, the pressure distribution on the rotor inner wall is obtained as

(27) $$\begin{array}{cc} p(a,\theta ) & =\frac{1}{2}\rho \Omega^{2}\left(a^{2}-b^{2}\right)\\ & +\frac{1}{2}\delta \rho a\omega^{2}\frac{\sigma_{1}^{2}+2\Omega \sigma_{1}-\Omega^{2}}{\kappa \sigma_{1}^{2}+2\Omega \sigma_{1}-\Omega^{2}}\cos(\sigma_{1}t+\theta )\\ & +\frac{1}{2}\delta \rho a\omega^{2}\frac{\sigma_{2}^{2}+2\Omega \sigma_{2}-\Omega^{2}}{\kappa \sigma_{2}^{2}+2\Omega \sigma_{2}-\Omega^{2}}\cos(\sigma_{2}t+\theta ) \end{array}$$

The *x*- and *y*-components of the resultant force exerted by the fluid on the rotor cylinder are given by

(28) $$\left\{\begin{array}{l} F_{x}=\int_{0}^{2\pi }p(a,\theta )\cos\left(\Omega t+\theta \right)Lad\theta =\frac{1}{2}\delta m_{c}\omega^{2}\cos\omega t\left(f_{1}+f_{2}\right)\\ F_{y}=\int_{0}^{2\pi }p(a,\theta )\sin(\Omega t+\theta )Lad\theta =\frac{1}{2}\delta m_{c}\omega^{2}\sin\omega t\left(-f_{1}+f_{2}\right) \end{array}\right.$$

where $m_{c}=\rho L\pi a^{2}$ denotes the mass of the liquid filling the rotor cylinder and the explicit forms of $f_{1}$ and $f_{2}$ are given as follows:

(29) $$\left\{\begin{array}{l} f_{1}=\frac{\sigma_{1}^{2}+2\Omega \sigma_{1}-\Omega^{2}}{\kappa \sigma_{1}^{2}+2\Omega \sigma_{1}-\Omega^{2}}\\ f_{2}=\frac{\sigma_{2}^{2}+2\Omega \sigma_{2}-\Omega^{2}}{\kappa \sigma_{2}^{2}+2\Omega \sigma_{2}-\Omega^{2}} \end{array}\right.$$

A mass ratio parameter is introduced as $\mu =m_{c}/m_{R}$, where *b* denotes the mass of the dry rotor. Equation (28) can be rewritten as

(30) $$\left[\begin{array}{l} F_{x}\\ F_{y} \end{array}\right]=\frac{1}{2}\delta \mu m_{R}\omega^{2}\left[\begin{array}{cc} f_{1}+f_{2} & 0\\ 0 & f_{2}-f_{1} \end{array}\right]\left[\begin{array}{c} \cos\omega t\\ \sin\omega t \end{array}\right]$$

### 2.2. Structural Dynamics

The rotor equations of motion are given by

(31) $$\left[\begin{array}{ll} m_{R} & \\ & m_{R} \end{array}\right]\left[\begin{array}{l} {\ddot{x}}_{c}\\ {\ddot{y}}_{c} \end{array}\right]+\left[\begin{array}{ll} c & \\ & c \end{array}\right]\left[\begin{array}{l} {\dot{x}}_{c}\\ {\dot{y}}_{c} \end{array}\right]+\left[\begin{array}{ll} k & \\ & k \end{array}\right]\left[\begin{array}{l} x_{c}\\ y_{c} \end{array}\right]=\left[\begin{array}{l} F_{x}\\ F_{y} \end{array}\right]$$

where $m_{R}$ denotes the dry-rotor mass, c denotes the external damping coefficient, and k denotes the combined stiffness. By solving Equation (31), the steady-state forced-vibration response of the partially liquid-filled rotor is obtained as follows:

(32) $$\left\{\begin{array}{l} x_{c}\left(t\right)=\frac{{\tilde{F}}_{x}}{m_{R}\omega_{n}^{2}\sqrt{{\left(1-G^{2}\right)}^{2}+{\left(2\xi G\right)}^{2}}}\cos\left(\omega t-\varphi \right)\\ y_{c}\left(t\right)=\frac{{\tilde{F}}_{y}}{m_{R}\omega_{n}^{2}\sqrt{{\left(1-G^{2}\right)}^{2}+{\left(2\xi G\right)}^{2}}}\sin\left(\omega t-\varphi \right) \end{array}\right.$$

where

(33) $$\begin{array}{l} \left\{\begin{array}{l} {\tilde{F}}_{x}=\frac{1}{2}\delta m_{c}\omega^{2}\left(f_{1}+f_{2}\right)\\ {\tilde{F}}_{y}=\frac{1}{2}\delta m_{c}\omega^{2}\left(-f_{1}+f_{2}\right) \end{array}\right. \end{array}$$

(34) $$\omega_{n}=\sqrt{\frac{k}{m_{R}},}\;G=\frac{\omega }{\omega_{n}},\;\;\xi =\frac{c}{2\sqrt{km_{R}}},\;\;\varphi =\arctan\frac{2\xi G}{1-G^{2}}$$

Substituting Equation (33) into Equation (32) and introducing the dimensionless variables yields

(35) $$\left\{\begin{array}{l} {\bar{x}}_{c}\left(t\right)=\frac{1}{2}\mu G^{2}\frac{\left(\frac{{\left(S+G\right)}^{2}+2S\left(S+G\right)-{\left(S\right)}^{2}}{\kappa {\left(S+G\right)}^{2}+2S\left(S+G\right)-{\left(S\right)}^{2}}+\frac{{\left(S-G\right)}^{2}+2S\left(S-G\right)-{\left(S\right)}^{2}}{\kappa {\left(S-G\right)}^{2}+2S\left(S-G\right)-{\left(S\right)}^{2}}\right)}{\sqrt{{\left(1-G^{2}\right)}^{2}+{\left(2\xi G\right)}^{2}}}\cos\left(\omega t-\varphi \right)\\ {\bar{y}}_{c}\left(t\right)=\frac{1}{2}\mu G^{2}\frac{\left(-\frac{{\left(S+G\right)}^{2}+2S\left(S+G\right)-{\left(S\right)}^{2}}{\kappa {\left(S+G\right)}^{2}+2S\left(S+G\right)-{\left(S\right)}^{2}}+\frac{{\left(S-G\right)}^{2}+2S\left(S-G\right)-{\left(S\right)}^{2}}{\kappa {\left(S-G\right)}^{2}+2S\left(S-G\right)-{\left(S\right)}^{2}}\right)}{\sqrt{{\left(1-G^{2}\right)}^{2}+{\left(2\xi G\right)}^{2}}}\sin\left(\omega t-\varphi \right) \end{array}\right.$$

where γ is the fluid-fill ratio, and

(36) $${\bar{x}}_{c}=\frac{x_{c}}{\delta };{\bar{y}}_{c}=\frac{y_{c}}{\delta };S=\frac{\Omega }{\omega_{n}};\gamma =\frac{b}{a};\kappa =\frac{1+\gamma^{2}}{1-\gamma^{2}}$$

## 3. Vibration Response of Partially Liquid-Filled Rotors

### 3.1. Dynamic Responses of the Partially Liquid-Filled Rotor System

A numerical simulation is conducted in this section to investigate the steady-state dynamic response of the partially liquid-filled rotor system, based on the dynamical model established in the previous section. The baseline parameters are as follows: rotormass ratio μ=0.5; liquid-fill ratio γ=0.5; nondimensional rotational frequency S=4; and nondimensional excitation frequency G=2.

**Figure 3:**
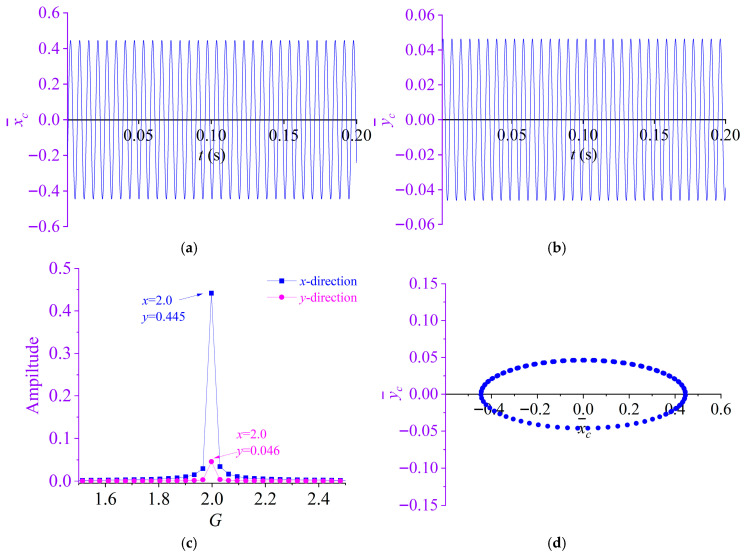
Dynamic responses of the partially liquid-filled rotor system: (**a**) Displacement time histories in the *x*-directions; (**b**) displacement time histories in the *y*-directions; (**c**) response amplitude–frequency characteristic curve; (**d**) rotor centroid orbit.

By substituting the prescribed baseline parameters into Equation (35) and evaluating the response at the rotor geometric center, the dynamic responses of the partially liquid-filled rotor system are obtained, as shown in Figure 3. Figure 3a,b shows the displacement time histories in the *x*- and *y*-directions, respectively. Figure 3c presents the corresponding displacement spectra, and Figure 3d illustrates the orbit of the rotor center.

As shown in Figure 3a,b, the displacement responses of the rotor in the *x*- and *y*-directions exhibit a stable harmonic pattern in the time domain. The corresponding spectra in Figure 3c indicate that both responses are dominated by a single synchronous component, whose dominant frequency coincides with that of the lateral excitation acting on the partially liquid-filled rotor. The maximum amplitude in the *x*-direction is 0.445, whereas that in the *y*-direction is 0.046. In Figure 3d, the rotor centroid orbit forms an annular trajectory. These results demonstrate that a lateral excitation applied in the *x*-direction induces a coupled vibration response in the *y*-direction, with the *x*-direction amplitude exceeding the y-direction amplitude.

As indicated by Equation (35), the rotor centroid *c* executes simple harmonic motions in two mutually orthogonal directions with a common angular frequency *ω*. By eliminating the time variable *t*, the governing equation for the trajectory (orbit) of the rotor centroid can be obtained.

(37) $$\frac{{\bar{x}}_{c}^{2}}{A_{x}^{2}}+\frac{{\bar{y}}_{c}^{2}}{A_{y}^{2}}=1$$

where *A_x_* and *A_y_* denote the amplitude coefficients of the responses in the *x*- and *y*-directions, respectively; the corresponding semi-axis lengths of the orbit are |*A_x_*| and |*A_y_*|.

(38) $$A_{x}=\frac{1}{2}\mu G^{2}\frac{F_{+}+F_{-}}{\sqrt{{\left(1-G^{2}\right)}^{2}+{\left(2\xi G\right)}^{2}}},\;A_{y}=\frac{1}{2}\mu G^{2}\frac{-F_{+}+F_{-}}{\sqrt{{\left(1-G^{2}\right)}^{2}+{\left(2\xi G\right)}^{2}}}$$

where

(39) $$\left\{\begin{array}{l} F_{+}=\frac{{\left(S+G\right)}^{2}+2S(S+G)-S^{2}}{\kappa {\left(S+G\right)}^{2}+2S(S+G)-S^{2}}\\ F_{-}=\frac{{\left(S-G\right)}^{2}+2S(S-G)-S^{2}}{\kappa {\left(S-G\right)}^{2}+2S(S-G)-S^{2}} \end{array}\right.$$

As implied by Equation (37), the rotor-center orbit is elliptical, and the resulting whirl motion can be viewed as the superposition of rotor spin and center precession about the undeformed shaft centerline.

### 3.2. Effects of Parameters on the Dynamic Characteristics

Equation (35) shows that the system response is governed by several key parameters, including the mass ratio *μ*, the fill ratio *γ*, the nondimensional rotational frequency *S*, and the nondimensional excitation frequency *G*. This section presents a parametric analysis to quantify how variations in these parameters affect the system’s dynamic characteristics.

#### 3.2.1. Mass Ratio

To assess the influence of the mass ratio, the mass ratio *μ* is set to 0.1,0.3, 0.5, 0.7 and 0.9, while all other parameters are kept identical to those in Section 3.1.

**Figure 4:**
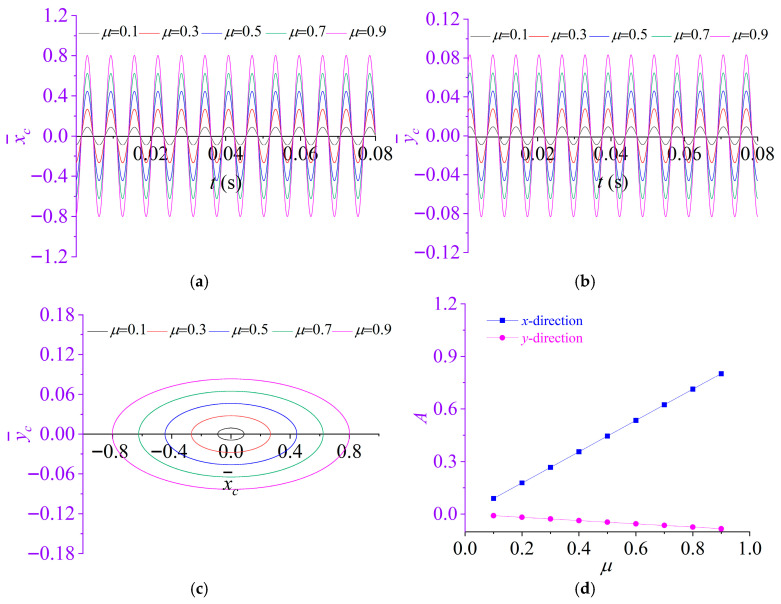
Effect of mass ratio *μ* on the dynamic response of rotor system: (**a**) displacement time histories in the *x*-directions; (**b**) displacement time histories in the *y*-directions; (**c**) rotor centroidorbit; (**d**) amplitude coefficients of the responses in the *x*- and *y*-directions.

The vibration characteristics of the partially liquid-filled rotor are then evaluated. Figure 4 summarizes the time-domain displacement responses of the rotor in the *x*- and *y*-directions for the different mass ratios, along with the corresponding rotor centroid orbit and amplitude coefficients of the responses. It can be seen that the mass ratio *μ* exerts a pronounced influence on the steady-state response amplitudes of the system. As *μ* increases from 0.1 to 0.9, the time-domain responses of the rotor in the *x*- and *y*-directions remain stable and harmonic, and the dominant response frequency is essentially insensitive to *μ*. In contrast, the overall vibration level increases with *μ*, with the *x*-direction amplitude consistently exceeding that in the *y*-direction. As indicated in Figure 4c, the corresponding rotor centroid orbits form a family of concentric ellipses that progressively expand outward as *μ* increases; meanwhile, the major-to-minor axis ratio gradually increases, implying that *μ* affects not only the response magnitude but also the orbit geometry. Moreover, Figure 4d shows that the amplitude coefficient *A_x_* increases approximately monotonically with *μ*, whereas *A_y_* exhibits only minor variations around zero. This observation suggests that the response is dominated by the primary vibration in the *x*-direction, while the cross-coupled vibration in the *y*-direction remains comparatively weak.

#### 3.2.2. Liquid-Fill Ratio

To investigate the influence of the liquid-fill ratio on the vibration response of the rotor system, a parametric study is carried out by varying the liquid-fill ratio *γ*. Specifically, *γ* is assigned values of 0, 0.1, 0.2, 0.3 and 0.4 while all other parameters are held constant at those specified in Section 3.1.

**Figure 5:**
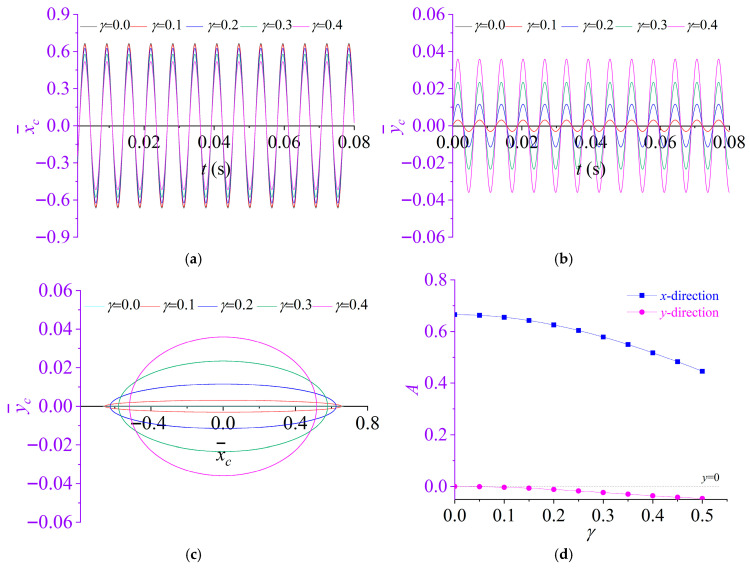
Effect of liquid-fill ratio *γ* on the dynamic response of rotor system: (**a**) displacement time histories in the *x*-directions; (**b**) displacement time histories in the *y*-directions; (**c**) rotor centroid orbit; (**d**) amplitude coefficients of the responses in the *x*- and *y*-directions.

The dynamic responses of the partially liquid-filled rotor system are then computed and compared across the considered fill ratios. Figure 5 demonstrates that the liquid-fill ratio *γ* significantly modulates both the response amplitudes and the rotor centroid orbit of the rotor system. For the fully filled configuration (*b* = 0), *γ* = 0, under which the rotor exhibits a purely one-dimensional vibration along the *x*- direction and the *y*-direction amplitude coefficient vanishes (*A_y_* = 0), indicating the absence of cross-coupled motion. As *γ* increases, the time-domain responses in the *x*- and *y*-directions remain stable and harmonic, while the dominant response frequency remains essentially unchanged, suggesting that *γ* primarily affects the response magnitude rather than the characteristic frequency. More importantly, *γ* alters the orbit geometry: the rotor centroid orbitevolves from a degenerate line segment at *γ* = 0 to a closed elliptical orbit for *γ* > 0, indicating a transition from uncoupled to weakly coupled lateral motion. Consistent with these observations, Figure 5d shows that the *x*-direction amplitude coefficient *A_x_* decreases monotonically with increasing *γ*, whereas *A_y_* becomes nonzero but remains small in magnitude, implying that increasing *γ* suppresses the primary *x*-direction vibration while inducing only a weak coupled response in the *y*-direction.

#### 3.2.3. Rotational Frequency *S*

**Figure 6:**
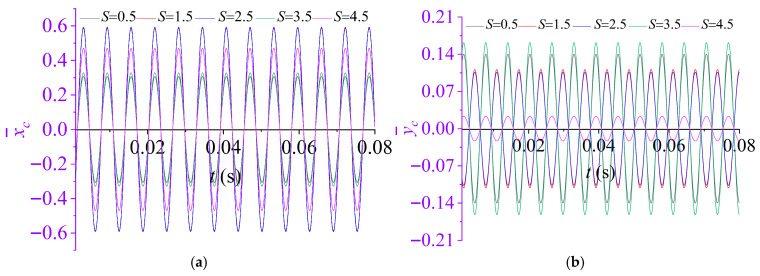

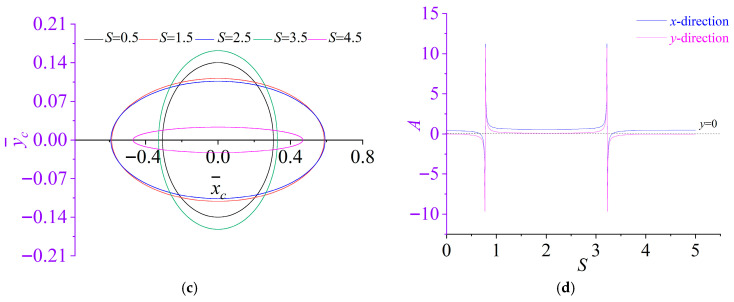
Effect of nondimensional rotational frequency *S* on the dynamic response of rotor system: (**a**) displacement time histories in the *x*-directions; (**b**) displacement time histories in the *y*-directions; (**c**)rotor centroid orbit; (**d**) amplitude coefficients of the responses in the *x*- and *y*-directions.

To quantify the effect of the nondimensional rotational frequency *S* on the vibration response of the partially liquid-filled rotor system, a parametric study is conducted in which *S* is varied while all other parameters are held fixed at the values specified in Section 3.1. Specifically, *S* is set to 0.5, 1.5, 2.5, 3.5 and 4.5. The corresponding dynamic responses are computed and compared across the considered cases, and the results are summarized in Figure 6.

Figure 6 indicates that the nondimensional rotational frequency *S* exerts a significant influence on the steady-state response amplitudes as well as the rotor centroid orbitof the partially liquid-filled rotor system. As shown in Figure 6a,b, the time-domain responses of the rotor mass center in the *x*- and *y*-directions remain stable and harmonic over the range of *S* considered, and the dominant spectral component is essentially independent of *S*. By contrast, the response amplitudes vary markedly with S and exhibit a distinctly non-monotonic dependence.

Furthermore, the amplitude–coefficient curves in Figure 6d reveal the presence of several “critical” intervals in the dependence of *A_x_* and *A_y_* on the nondimensional rotational frequency *S*. As *S* approaches these critical values, the amplitude coefficients increase abruptly, indicating pronounced resonant or near-resonant amplification of the system response. This behavior can be interpreted analytically from Equation (39): when

(40) $$S=G\left(1\pm \sqrt{\frac{1-\gamma^{2}}{2}}\right)$$

The denominator of $F_{-}$ tends to zero, leading to a singularity in the response function and, consequently, a resonance-type amplification. Accordingly, the corresponding rotational frequency is defined as the system’s critical rotational frequency. When *S* is sufficiently far from the critical value, the amplitude coefficients remain at relatively low levels and the system response is comparatively stable. By contrast, as S approaches the critical value, the response is markedly amplified and is accompanied by a pronounced change in the shaft-center orbit. Overall, *S* not only determines whether the system enters a resonant amplification regime, but also modifies the orbit geometry and the lateral coupling characteristics by regulating the relative magnitude of *A_x_* and *A_y_*.

#### 3.2.4. Excitation Frequency *G*

**Figure 7:**
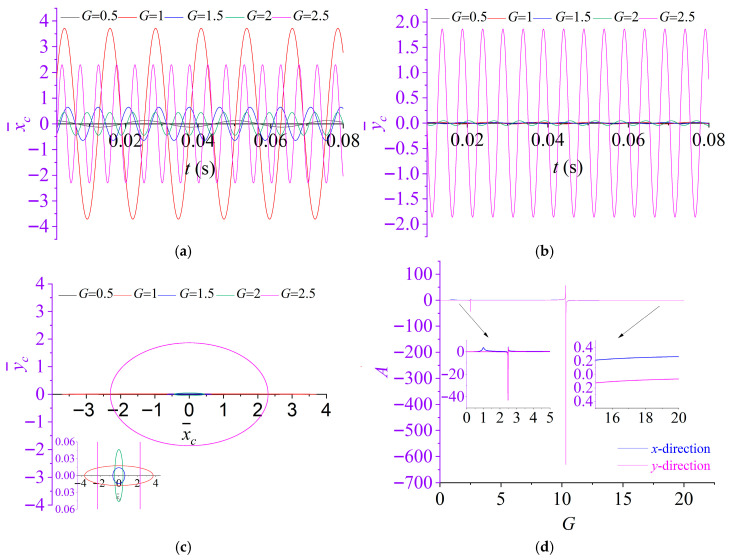
Effect of the dimensionless excitation frequency *G* on the dynamic response of rotor system: (**a**) displacement time histories in the *x*-directions; (**b**) displacement time histories in the *y*-directions; (**c**) rotor centroid orbit; (**d**) amplitude coefficients of the responses in the *x*- and *y*-directions.

To elucidate the effect of the dimensionless excitation frequency *G* on the rotor vibration response, a parametric investigation was performed by assigning *G* = 0.5, 1.0, 1.5, 2.0, and 2.5, while maintaining all remaining parameters consistent with those in Section 3.1. For each prescribed *G*, the governing equations were numerically integrated to obtain the steady-state dynamic responses. The computed results are presented in Figure 7.

Figure 7 indicates that the nondimensional excitation frequency *G* exerts a decisive influence on the steady-state dynamics of the partially liquid-filled rotor, affecting the response level, oscillation period, and rotor centroid orbit. As shown in Figure 7a,b, the responses of the rotor remain stable and harmonic across the investigated cases; nevertheless, the temporal scale of the motion varies with *G*. In particular, the oscillation frequency increases with *G*, leading to a corresponding reduction in the vibration period. Beyond this kinematic dependence, Figure 7d reveals a distinctly non-monotonic relationship between the amplitude coefficients and *G*, with sharp growth and peak-like amplification near specific frequencies, which is characteristic of resonant or near-resonant behavior. When *G* = 1, the excitation frequency coincides with the (normalized) natural frequency of the rotor system, resulting in a pronounced (primary) resonance and a substantial amplification of the response. In addition, resonance amplification is also triggered when *G* satisfies

(41) $$G=S{\left(1\pm \sqrt{\frac{1-\gamma^{2}}{2}}\right)}^{-1}$$

under which the response transfer function exhibits a singularity (i.e., the denominator tends to zero), causing the amplitude coefficients to escalate rapidly. Consistent with this amplification, the rotor centroidorbit in Figure 7c expands markedly and undergoes an evident geometric transformation in the resonant regime, reflecting substantial changes in the relative *x*- and *y*-direction response levels and the associated lateral coupling. Overall, *G* governs both the vibration time scale (frequency/period) and the onset of resonant regimes that dramatically intensify the response and reshape the orbit morphology.

This amplification behavior observed in Figure 6 and Figure 7 can also be interpreted analytically from Equations (29) and (31). When the nondimensional parameters *S* and *G* satisfy the critical condition given by Equation (42), the denominator of $f_{2}$ in Equation (29) tends to zero, leading to singular amplification of the analytical force coefficients and, consequently, of the response components $F_{x}$ and $F_{y}$ in Equation (31).

This analytical prediction is consistent with the classical solution reported by Tao and Zhang (1993) [51], who demonstrated that two critical frequencies exist for a given fill ratio and that the disturbance force exhibits unbounded growth near these conditions.

(42) $$\frac{G}{S}=\frac{1}{\left(1\pm \sqrt{\frac{1-\gamma^{2}}{2}}\right)}$$

Therefore, the present formulation correctly reproduces the critical-frequency mechanism and the associated resonance amplification behavior of partially liquid-filled rotors under lateral excitation.

It should be noted that this singular behavior is a characteristic of the linearized analytical model and represents the onset of resonance in an idealized system. In practical rotor systems, the response would remain bounded due to viscous dissipation, structural damping, and nonlinear effects.

## 4. Data-Driven Surrogate Modeling and Uncertainty Analysis

As demonstrated in Section 3, partially liquid-filled rotors (PLFRs) exhibit pronounced fluid–structure coupling, and their responses may undergo spike-like amplification in the vicinity of critical operating conditions. In practical engineering applications, key parameters—such as the fill ratio, effective damping, excitation level, and frequency-related dimensionless quantities—are inevitably subject to uncertainty due to manufacturing tolerances, assembly errors, operational variability, and fluctuations in fluid properties. Because the coupled dynamics can substantially amplify these perturbations and propagate them to the system response, deterministic predictions based solely on nominal parameter values are insufficient to reliably quantify vibration risk and stability margins. Therefore, this section develops a stochastic modeling and uncertainty analysis framework for PLFRs to probabilistically characterize the key inputs and to quantify the resulting variability in the dynamic responses, thereby enabling risk-informed assessment and robust design.

### 4.1. Surrogate Modeling Using Support Vector Machine and Backpropagation Neural Network

Efficient uncertainty propagation requires fast and reliable prediction of system responses across a wide range of uncertain parameters. While the analytical model provides accurate response evaluation, its direct use in large-scale stochastic simulations is computationally expensive. To improve computational efficiency, data-driven surrogate models are introduced to approximate the nonlinear input–output relationship. In this work, support vector machine (SVM) and backpropagation neural network (BPNN) models are employed due to their powerful nonlinear mapping capability, strong generalization performance, and proven effectiveness in surrogate modeling and uncertainty analysis of complex dynamical systems.

#### 4.1.1. Support Vector Machine Model

Support vector machine (SVM) is a supervised learning method grounded in statistical learning theory and structural risk minimization, and it has been widely used for nonlinear regression and surrogate modeling of complex dynamical systems. In the present study, the support vector regression (SVR) formulation of SVM is employed to construct a surrogate model that approximates the nonlinear mapping between uncertain input parameters and vibration response amplitudes of the partially liquid-filled rotor.

Consider a training dataset consisting of *N* samples:

(43) $$D={\left\{\left(x_{i},y_{i}\right)\right\}}_{i=1}^{N}$$

where $x_{i}\in R^{d}$ denotes the input vector containing uncertain parameters and $y_{i}\in R$ represents the corresponding vibration response amplitude. The SVR model seeks to construct a regression function of the form

(44) $$f(x)=w^{T}\phi (x)+b$$

where *ϕ*(*x*) is a nonlinear mapping from the input space to a high-dimensional feature space, w is the weight vector, and *b* is the bias term.

To determine the optimal regression function, SVR solves the following optimization problem based on the principle of structural risk minimization:

(45) $$\min_{w,b,\xi_{i},\xi_{i}^{*}}\frac{1}{2}\|w{\|}^{2}+C\sum_{i=1}^{N}\left(\xi_{i}+\xi_{i}^{*}\right)$$

Subject to

(46) $$\begin{array}{l} y_{i}-w^{T}\phi (x_{i})-b\leq \varepsilon +\xi_{i}\\ w^{T}\phi (x_{i})+b-y_{i}\leq \varepsilon +\xi_{i}^{*}\\ \xi_{i},\xi_{i}^{*}\geq 0 \end{array}$$

where C is the regularization parameter controlling the trade-off between model complexity and training error, ε defines the insensitive loss function, and $\xi_{i}$ and $\xi_{i}^{*}$ are slack variables representing prediction errors.

By introducing Lagrange multipliers and solving the dual optimization problem, the regression function can be expressed as

(47) $$f(x)=\sum_{i=1}^{N}\left(\alpha_{i}-\alpha_{i}^{*}\right)K\left(x_{i},x\right)+b$$

where $\alpha_{i},\alpha_{i}^{*}$ are Lagrange multipliers and $K\left(x_{i},x\right)$ is the kernel function.

In this study, the radial basis function (RBF) kernel is employed due to its superior capability in modeling highly nonlinear relationships:

(48) $$K(x_{i},x_{j})=\exp\left(-\gamma {\left\|x_{i}-x\right\|}^{2}\right)$$

where γ is the kernel parameter controlling the width of the Gaussian kernel.

The final SVR surrogate model establishes a nonlinear mapping between uncertain input parameters and vibration response amplitudes, enabling efficient and accurate prediction of system responses for large-scale uncertainty propagation. Compared with the analytical model, the SVR surrogate significantly reduces computational cost while maintaining high prediction accuracy, making it well suited for stochastic analysis and sensitivity evaluation of partially liquid-filled rotor systems.

#### 4.1.2. Backpropagation Neural Network Model

The backpropagation neural network (BPNN) is a feedforward artificial neural network trained using the error backpropagation algorithm. Due to its strong nonlinear approximation capability and universal function approximation property, BPNN has been widely applied in surrogate modeling and uncertainty quantification of complex engineering systems. In this study, a BPNN surrogate model is constructed to approximate the nonlinear relationship between uncertain input parameters and vibration response amplitudes of the partially liquid-filled rotor.

**Figure 8:**
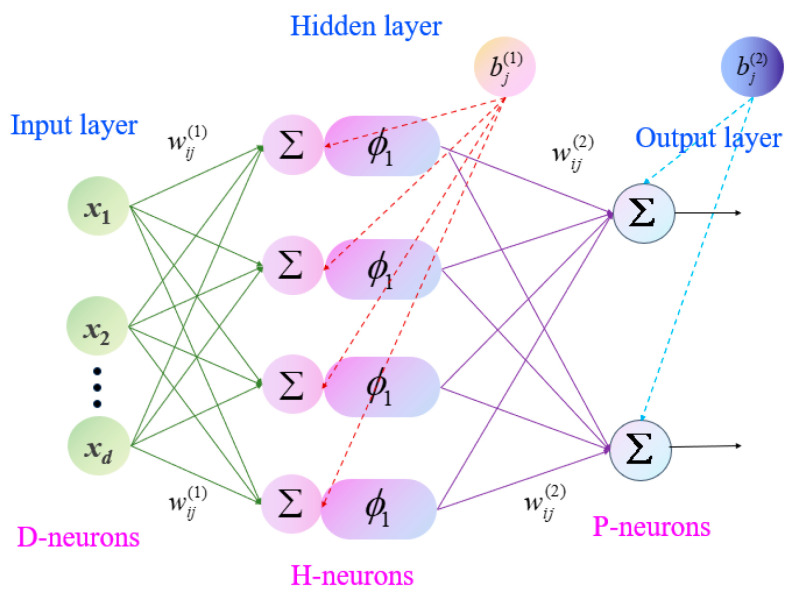
Topological structure of the backpropagation neural network (BPNN) surrogate model.

A typical backpropagation neural network (BPNN) comprises an input layer, one or more hidden layers, and an output layer. The architecture of the three-layer BPNN considered in this study is illustrated in Figure 8. The input vector can be expressed as

(49) $$X={\left[x_{1},x_{2},\ldots ,x_{d}\right]}^{T}$$

where *d* denotes the number of uncertain input parameters. The output of the hidden layer neuron *j* is given by

(50) $$h_{j}=\phi \left(\sum_{i=1}^{d}w_{ij}^{\left(1\right)}x_{i}+b_{j}^{\left(1\right)}\right)$$

where $w_{ij}^{\left(1\right)}$ represents the weight connecting input neuron *i* to hidden neuron *j*, $b_{j}^{\left(1\right)}$ is the bias of hidden neuron *j*, and ϕ(·) is the activation function.

Common activation functions include the sigmoid function

(51) $$\phi (z)=\frac{1}{1+e^{-z}}$$

or the hyperbolic tangent function

(52) ϕ(z)=tanh(z)

The output layer produces the predicted response as

(53) $$\hat{y}=\sum_{j=1}^{H}w_{ij}^{\left(2\right)}h_{j}+b^{\left(2\right)}$$

where *H* is the number of hidden neurons, $w_{j}^{\left(2\right)}$ denotes the weight connecting hidden neuron *j* to the output neuron, and $b^{\left(2\right)}$ is the output bias. The network is trained by minimizing the mean squared error (MSE) between the predicted and target outputs:

(54) $$E=\frac{1}{N}\sum_{i=1}^{N}{\left(y_{i}-{\hat{y}}_{i}\right)}^{2}$$

where *N* is the number of training samples.

The weights and biases are updated iteratively using the gradient descent-based backpropagation algorithm:

(55) $$w^{\mathrm{new}\;}=w^{\mathrm{old}\;}-\eta \frac{\partial E}{\partial w}$$

where *η* is the learning rate.

Through iterative forward propagation and backward error propagation, the BPNN learns the nonlinear mapping between uncertain input parameters and vibration responses. Once trained, the BPNN surrogate model enables efficient prediction of system responses with significantly reduced computational cost compared to direct analytical evaluation. This makes it particularly suitable for large-scale uncertainty propagation and sensitivity analysis of partially liquid-filled rotor systems.

### 4.2. Surrogate Model Validation and Comparison

#### 4.2.1. Definition of Uncertain Input Parameters and Probability Distributions

In partially liquid-filled rotor (PLFR) systems, several physical and operational parameters exhibit inherent uncertainties due to manufacturing tolerances, assembly variations, and operating condition fluctuations. Among these parameters, the mass ratio μ, liquid-fill ratio γ, dimensionless rotational frequency *S*, and dimensionless excitation frequency *G* are selected as the primary uncertain inputs in this study. These parameters play a dominant role in governing the coupled fluid–structure dynamics and directly influence the resonance characteristics, energy transfer mechanisms, and amplitude amplification behavior of the system. In particular, variations in the fill ratio affect the strength of fluid–structure coupling, while the dimensionless frequency parameters determine the proximity to resonance conditions. Consequently, even small perturbations in these parameters may lead to significant changes in vibration amplitudes, especially near critical operating regions.

The uncertain input vector is defined as

(56) $$X={\left[\mu ,\;\gamma \;,\;\;S,\;G\right]}^{T}$$

where each parameter is modeled as an independent random variable following a normal distribution:

(57) $$X_{i}\sim N\left(\mu_{i},\sigma_{i}^{2}\right),\;i=1,2,3,4$$

where $\mu_{i}$ and $\sigma_{i}$ denote the mean and standard deviation, respectively. The standard deviation is defined using a prescribed coefficient of variation (CV),

(58) $$\sigma_{i}=CV\cdot \mu_{i}$$

This probabilistic representation captures the realistic variability of system parameters while ensuring physically meaningful perturbations around their nominal values.

It is important to note that the objective of the present uncertainty analysis is not to predict the full time-domain response, but rather to evaluate the extreme values of the steady-state vibration amplitudes in the lateral directions. This approach is motivated by the fact that the peak vibration amplitude represents the most critical indicator of system stability, structural integrity, and operational safety. Compared with full time-domain prediction, focusing on response extrema significantly reduces computational cost while preserving the key dynamic characteristics relevant to engineering design and reliability assessment. Moreover, extreme response metrics are directly associated with resonance amplification and potential failure risks, making them more suitable for uncertainty quantification and sensitivity analysis of nonlinear rotor systems.

Based on the defined probabilistic input model, surrogate models are subsequently employed to efficiently propagate parameter uncertainties and quantify their effects on the extreme vibration responses.

#### 4.2.2. Surrogate-Assisted Uncertainty Quantification Framework

**Figure 9:**
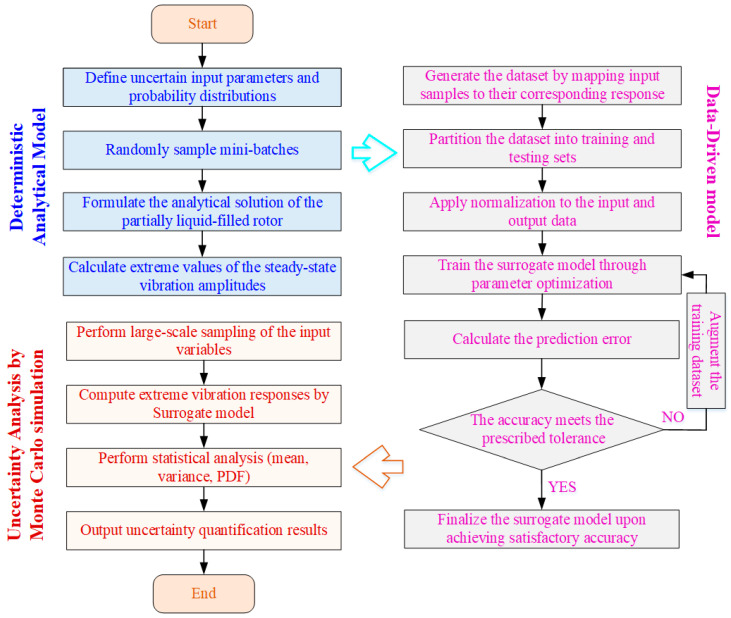
Flowchart of surrogate-assisted uncertainty quantification for partially liquid-filled rotor systems.

The overall framework integrating deterministic analytical modeling, data-driven surrogate modeling, and Monte Carlo-based uncertainty quantification is illustrated in Figure 9. The process begins with the definition of uncertain input parameters and their associated probability distributions. Random sampling is performed to generate input realizations, and the analytical solution of the partially liquid-filled rotor system is employed to compute the corresponding extreme values of steady-state vibration amplitudes. These input–output mappings form the dataset for surrogate model construction.

The generated dataset is subsequently divided into training and testing subsets and normalized to ensure numerical robustness and improved convergence. The surrogate model is trained through parameter optimization, and its predictive capability is rigorously assessed using error-based accuracy metrics. If the required accuracy is not achieved, additional samples are incorporated to enrich the training dataset and enhance model fidelity. This iterative process continues until the surrogate model satisfies the prescribed accuracy requirements.

Once validated, the surrogate model is employed to perform large-scale Monte Carlo simulations efficiently. The extreme vibration responses are rapidly evaluated, and statistical characteristics, including the mean, variance, and probability density function, are obtained to quantify the stochastic behavior of the rotor system. This surrogate-assisted framework significantly reduces computational cost while maintaining high prediction accuracy, enabling efficient and reliable uncertainty quantification.

#### 4.2.3. Comparative Validation of SVM and BPNN Models

To ensure the reliability of the surrogate-based uncertainty analysis, the predictive accuracy of the support vector machine (SVM) and backpropagation neural network (BPNN) models is systematically evaluated. This subsection first defines the uncertain input parameters and their probabilistic characteristics, followed by the introduction of accuracy evaluation metrics. Finally, the predictive performances of the two surrogate models are compared quantitatively and visually.

Table 1 lists the adopted uncertainty description for each parameter of partially liquid-filled rotor system. All parameters are assigned a coefficient of variation of 0.01, representing small but realistic uncertainty levels in engineering applications. These probabilistic descriptions provide the basis for generating training and testing datasets used for surrogate model construction and validation.

To quantitatively assess the predictive accuracy of the surrogate models, two widely used statistical metrics are employed: the mean absolute percentage error (MAPE) and the coefficient of determination (*R*^2^).

MAPE measures the relative prediction error and is defined as

(59) $$\mathrm{MAPE}=\frac{1}{N}\sum_{i=1}^{N}\left|\frac{y_{i}^{\mathrm{true}\;}-y_{i}^{\mathrm{pred}\;}}{y_{i}^{\mathrm{true}\;}}\right|\times 100\%$$

where $y_{i}^{\mathrm{true}\;}$ is the true value obtained from the analytical model, $y_{i}^{\mathrm{pred}\;}$ is the predicted value from the surrogate model, and *N* is the number of testing samples.

Coefficient of Determination (*R*^2^)

The coefficient of determination quantifies how well the surrogate model explains the variance of the true data and is defined as

(60) $$R^{2}=1-\frac{\sum_{i=1}^{N}{\left(y_{i}^{\mathrm{true}\;}-y_{i}^{\mathrm{pred}\;}\right)}^{2}}{\sum_{i=1}^{N}{\left(y_{i}^{\mathrm{true}\;}-{\bar{y}}^{\mathrm{true}\;}\right)}^{2}}$$

where

(61) $${\bar{y}}^{\mathrm{true}}=\frac{1}{N}\sum_{i=1}^{N}y_{i}^{\mathrm{true}}$$

An *R*^2^ value closer to 1 indicates higher prediction accuracy and better agreement between predicted and true values.

**Figure 10:**
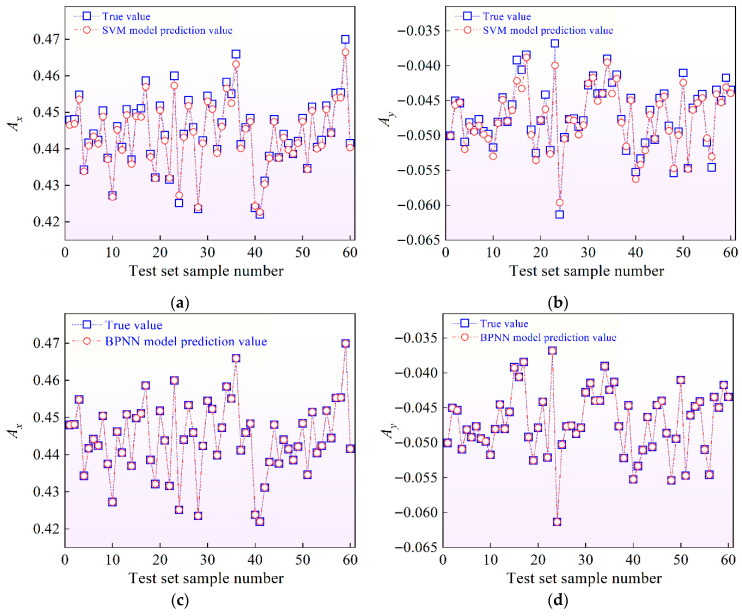
Validation of the SVM and BPNN surrogate models through comparison with analytical solutions for representative testing samples: (**a**) SVM prediction of response *A_x_*; (**b**) SVM prediction of response *A_y_*; (**c**) BPNN prediction of response *A_x_*; (**d**) BPNN prediction of response *A_y_*.

Figure 10 presents a comparison between the analytical solutions and the predictions obtained from the SVM and BPNN surrogate models for representative testing samples. It can be observed that both surrogate models successfully capture the overall trend of the analytical responses in both the *A_x_* and *A_y_* However, the BPNN model exhibits noticeably better agreement with the analytical solutions across the entire testing range. The predicted values obtained from the BPNN model show smaller deviations and closer overlap with the reference curves, indicating enhanced nonlinear mapping capability and improved generalization performance. This superior predictive consistency is evident in both the horizontal (*A_x_*) and vertical (*A_y_*) vibration responses.

The quantitative comparison of the two surrogate models is summarized in Table 2, where both the mean values and standard deviations of the evaluation metrics are reported, statistically derived from multiple independent runs of both surrogate models. As shown in the table, the BPNN model exhibits consistently lower mean absolute percentage errors (MAPEs) than the SVM model in both response directions, along with smaller corresponding standard deviations. This indicates that the BPNN surrogate not only achieves higher prediction accuracy but also demonstrates better stability and consistency across the test samples. In addition, the coefficients of determination (*R*^2^) of the BPNN model are closer to unity and show lower variability compared with those of the SVM model, further confirming its superior goodness of fit. Overall, these statistical results demonstrate that the BPNN surrogate provides more accurate and robust predictions than the SVM model in both directions, making it more suitable for subsequent uncertainty quantification and large-scale probabilistic analysis.

### 4.3. Uncertainty Propagation and Sensitivity Analysis of Maximum Amplitude Based on BPNN-Assisted Monte Carlo Simulation

#### 4.3.1. Statistical Distribution of Maximum Amplitudes Under Uncertainty

Using the trained BPNN surrogate model, large-scale Monte Carlo simulations were performed to efficiently predict the system responses under uncertainty. The uncertain parameters were modeled as random variables with coefficients of variation (CVs) ranging from 0.01 to 0.05. For each CV level, sufficient random samples were generated and propagated through the surrogate model to obtain the maximum vibration amplitudes in the *x*- and *y*-directions.

**Figure 11:**
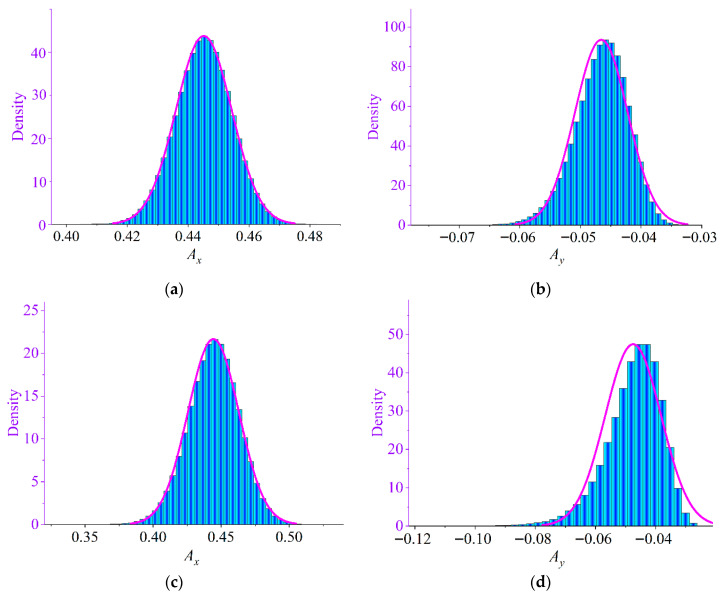
Histogram of the maximum amplitude distribution of the partially liquid-filled rotor system: (**a**) CV = 0.01, *A_x_*; (**b**) CV = 0.01, *A_y_*; (**c**) CV = 0.02, *A_x_*; (**d**) CV = 0.02, *A_y_*.

Figure 11 illustrates the probability density histograms of the maximum amplitudes for representative cases with CV = 0.01 and CV = 0.02. It can be observed that the maximum amplitude in the *x*-direction approximately follows a symmetric distribution resembling a Gaussian profile, indicating relatively stable stochastic behavior. In contrast, the amplitude distribution in the *y*-direction exhibits noticeable skewness, particularly under higher uncertainty levels. The distribution is asymmetric with a longer tail extending toward larger amplitude values, indicating an increased probability of extreme vibration responses.

The results indicate that although the input random parameters are normally distributed, the resulting maximum amplitude of the partially liquid-filled rotor system does not necessarily follow a normal distribution, due to the nonlinear propagation of uncertainty through the system dynamics.

The detailed statistical parameters of the maximum amplitudes for CVs ranging from 0.01 to 0.05 are summarized in Table A1 in Appendix A, including minimum value, maximum value, mean value, and standard deviation. As shown in Table A1, when the coefficient of variation is small (CV ≤ 0.02), both *x*- and *y*-direction amplitudes remain within relatively narrow ranges, indicating stable system dynamics. However, as the uncertainty level increases beyond CV = 0.03, the maximum amplitudes increase dramatically. For example, in the *x*-direction and *y*-direction, the maximum amplitude rises sharply, and the standard deviation also increases significantly, reflecting stronger variability and instability.

This phenomenon indicates that uncertainty can trigger excessive vibration amplification. The occurrence of abnormally large amplitudes suggests that the system may approach or enter resonance conditions due to parameter uncertainty. Therefore, it is critical to strictly control the uncertainty level of key system parameters in engineering design to avoid excessive vibration and ensure operational safety and reliability.

#### 4.3.2. Sensitivity Analysis Based on Spearman Rank Correlation

To further identify the relative importance of uncertain parameters, sensitivity analysis was performed using the Spearman rank correlation coefficient, which quantifies the monotonic relationship between input parameters and output responses.

The Spearman coefficient is defined as

(62) $$\rho_{i}=\frac{\sum_{k=1}^{N}\left(R\left(X_{i,k}\right)-\bar{R\left(X_{i}\right)}\right)\left(R\left(Y_{k}\right)-\bar{R(Y)}\right)}{\sqrt{\sum_{k=1}^{N}{\left(R\left(X_{i,k}\right)-\bar{R\left(X_{i}\right)}\right)}^{2}}\sqrt{\sum_{k=1}^{N}{\left(R\left(Y_{k}\right)-\bar{R(Y)}\right)}^{2}}}$$

where *X_i_* represents the *i*-th uncertain parameter, *Y* represents the maximum amplitude response, *R*(·) denotes rank transformation, *N* is the number of Monte Carlo samples, and the magnitude of *ρ*_i_ indicates the sensitivity strength of the parameter.

In this study, the absolute value of the Spearman coefficient |*ρ_i_*| is used for sensitivity visualization, because the absolute value directly reflects the sensitivity magnitude of each parameter, regardless of whether the correlation is positive or negative. A larger absolute value indicates a stronger influence on the system response, while the sign only reflects the direction of correlation. Since the primary objective of sensitivity analysis is to identify the most influential parameters, the absolute values are adopted for clearer comparison.

**Figure 12:**
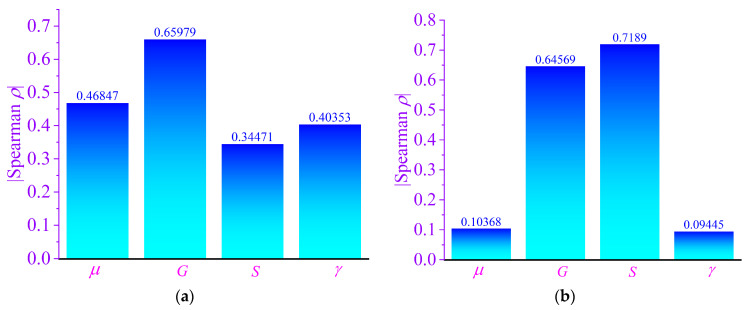
Sensitivity of the maximum amplitude: (**a**) Spearman Sensitivity for *A_x_*; (**b**) Spearman sensitivity for *A_y_*.

Figure 12 presents the sensitivity magnitudes of the uncertain parameters for the maximum amplitudes in both directions at CV = 0.01.

It can be observed that the dominant sensitive parameters differ between the *x*- and *y*-directions. In the *x*-direction, parameters *μ* and *G* exhibit the highest sensitivity levels, indicating that variations in these parameters significantly influence vibration amplitude. In contrast, in the *y*-direction, parameter *S* becomes the most influential factor, followed by *G*, while other parameters show relatively weaker effects.

This directional difference in sensitivity further explains the asymmetric and skewed distribution observed in the *y*-direction amplitude. The strong influence of specific parameters increases the likelihood of large amplitude excursions when uncertainty increases.

Overall, the uncertainty analysis reveals that the maximum amplitude in the *y*-direction exhibits skewed statistical characteristics and stronger sensitivity to parameter variations. When the coefficient of variation exceeds 0.02, the system may experience excessive vibration amplitudes due to uncertainty-induced resonance effects. Therefore, controlling parameter uncertainty is essential to ensure vibration stability and avoid resonance-related risks.

#### 4.3.3. Discussion

To further clarify the proposed model and its physical implications, a comparison with existing studies is presented as follows. The present results are generally consistent with previous studies on partially liquid-filled rotors, which have reported the fluid dynamic pressure and cross-coupled vibration behavior induced by fluid–structure interactions [47,52]. In particular, the coupling between orthogonal vibration directions observed in this study agrees well with the fluid-induced interaction mechanisms identified in analytical and stability analyses of liquid-filled rotor systems.

Furthermore, the amplification of vibration amplitude near specific excitation frequencies is consistent with the resonance characteristics reported in rotor–liquid interaction studies [14,51]. These studies have demonstrated that the redistribution of the internal liquid and the associated hydrodynamic forces can significantly modify the dynamic response and lead to critical conditions where the vibration amplitude increases rapidly.

In addition, the influence of liquid redistribution on vibration characteristics observed in this work is in line with the findings reported in fluid balancer and rotor–liquid coupling studies [53], where the internal fluid motion introduces additional dynamic forces that may either suppress or amplify rotor vibration depending on operating conditions.

Unlike most existing studies that primarily focus on deterministic response or stability boundaries, the present work further reveals that the maximum vibration amplitude exhibits a non-Gaussian and asymmetric distribution under parameter uncertainty, with a pronounced tail toward larger values. This phenomenon originates from the nonlinear mapping between uncertain parameters and system response, particularly under near-resonance conditions where small parameter variations can result in significant amplification.

From a physical perspective, the asymmetric response is attributed to the nonuniform redistribution of the internal liquid and the resulting unbalanced pressure field, which introduces cross-coupling effects between orthogonal vibration directions. The long-tailed distribution indicates an increased probability of extreme vibration responses, which is of particular importance for reliability assessment and cannot be captured by conventional deterministic analysis.

It should be noted that the present model is developed under the assumptions of small perturbation and inviscid flow. Therefore, the framework is primarily applicable to partially liquid-filled rotor systems operating under moderate vibration amplitudes and low-viscosity conditions. Effects such as viscous dissipation, strongly nonlinear free-surface deformation, and large-amplitude fluid motion are not considered, which may influence the response under more complex operating conditions.

## 5. Conclusions

This study developed a deterministic–data-driven framework for vibration prediction and uncertainty quantification of partially liquid-filled rotor systems subjected to lateral excitation. Based on small-perturbation flow theory, the liquid-induced feedback forces were derived and incorporated into the coupled rotor–liquid dynamic equations, yielding a closed-form steady-state solution. The results showed that lateral excitation in one direction induces coupled vibration in the orthogonal direction through fluid–structure interactions, leading to an elliptical whirl trajectory of the rotor center. In addition, the response characteristics were found to depend jointly on excitation frequency and rotor angular velocity, and two critical excitation frequencies were identified for a given angular velocity.

Using the analytical model, two surrogate models were constructed for uncertainty-aware response prediction. Comparative results showed that the BPNN model provides higher predictive accuracy than the SVM model for both *x*- and *y*-direction responses, indicating its stronger capability in approximating the nonlinear mapping of the coupled system. The uncertainty analysis further revealed that the maximum vibration amplitude does not necessarily follow a Gaussian distribution even when the input parameters are normally distributed, but may instead exhibit asymmetric response distributions with an increased probability of extreme amplitudes. Moreover, excessive response amplification may occur when the uncertainty level exceeds a certain threshold, highlighting the importance of controlling parameter variability in practical rotor design.

Overall, the present work contributes by establishing a physics-based analytical model for the forced response of partially liquid-filled rotors, proposing a deterministic–data-driven uncertainty quantification framework, and providing new physical insight into critical excitation conditions and non-Gaussian stochastic vibration characteristics. The proposed framework offers a reliable tool for probabilistic vibration assessment and uncertainty-informed design of partially liquid-filled rotor systems.

## Figures and Tables

**Table 1:** Characteristic of random variables of the partially liquid-filled rotor system.

Random Variables	Mean Values	CV
Mass ratio *μ*	0.5	0.01
Liquid-fill ratio γ	0.5	0.01
Nondimensional rotational frequency *S*	4	0.01
Nondimensional excitation frequency *G*	2	0.01

**Table 2:** Accuracy evaluation of BPNN and SVM surrogate models.

		BPNN		SVM	
		*A_x_*	*A_y_*	*A_x_*	*A_y_*
Mean	MAPE (%)	0.000345	0.019186	0.251410	1.626479
	*R* ^2^	0.999978	0.999989	0.974876	0.957189
Standard deviation	MAPE (%)	2.704 × 10^−5^	9.993 × 10^−5^	2.610 × 10^−4^	4.340 × 10^−4^
	*R* ^2^	2.676 × 10^−5^	9.219 × 10^−6^	1.643 × 10^−3^	4.381 × 10^−3^

## Data Availability

The original contributions presented in this study are included in the article. Further inquiries can be directed to the corresponding author.

## Appendix A

**Table A1:** Statistical parameters of the maximum vibration amplitude of the partially liquid-filled rotor system under different coefficients of variation.

		CV = 0.01	CV = 0.02	CV = 0.03	CV = 0.04	CV = 0.05
*A_x_*	Minimum amplitude	0.3958	0.3264	−0.2206	−90.3006	−158.7611
	Maximum amplitude	0.4895	0.5318	0.5777	28.0032	359.9398
	Mean value	0.4451	0.4443	0.4428	0.4396	0.4352
	Standard deviation	0.0091	0.0187	0.0294	0.1632	0.8169
*A_y_*	Minimum amplitude	−0.0766	−0.1486	−0.7112	−90.8051	−159.2150
	Maximum amplitude	−0.0316	−0.0216	−0.0156	27.5371	359.4900
	Mean value	−0.0465	−0.0475	−0.0493	−0.0528	−0.0578
	Standard deviation	0.00436	0.0092	0.0160	0.1597	0.8158
